# Burdock Fructooligosaccharide Attenuates High Glucose-Induced Apoptosis and Oxidative Stress Injury in Renal Tubular Epithelial Cells

**DOI:** 10.3389/fphar.2021.784187

**Published:** 2021-12-09

**Authors:** Mengru Ding, Zhiyan Tang, Wei Liu, Taili Shao, Pingchuan Yuan, Kaoshan Chen, Yuyan Zhou, Jun Han, Jing Zhang, Guodong Wang

**Affiliations:** ^1^ Drug Research and Development Center, School of Pharmacy, Wannan Medical College, Wuhu, China; ^2^ Anhui Provincial Engineering Laboratory for Screening and Re-evaluation of Active Compounds of Herbal Medicines, Anhui Provincial Engineering Research Center for Polysaccharide Drugs, Wuhu, China; ^3^ Anhui Province Key Laboratory of Active Biological Macromolecules, Wuhu, China; ^4^ Department of Nephrology, The First Affiliated Hospital, Yijishan Hospital of Wannan Medical College, Wuhu, China

**Keywords:** burdock fructooligosaccharide, NRK-52E cells, high glucose, apoptosis, oxidative stress

## Abstract

Hyperglycemia-induced apoptosis and oxidative stress injury are thought to play important roles in the pathogenesis of diabetic nephropathy (DN). Attenuating high glucose (HG)-induced renal tubular epithelial cell injury has become a potential approach to ameliorate DN. In recent years, burdock fructooligosaccharide (BFO), a water-soluble inulin-type fructooligosaccharide extracted from burdock root, has been shown to have a wide range of pharmacological activities, including antiviral, anti-inflammatory, and hypolipidemic activities. However, the role and mechanism of BFO in rat renal tubular epithelial cells (NRK-52E cells) have rarely been investigated. The present study investigated the protective effect of BFO on HG-induced damage in NRK-52E cells. BFO could protect NRK-52E cells against the reduced cell viability and significantly increased apoptosis rate induced by HG. These anti-oxidative stress effects of BFO were related to the significant inhibition of the production of reactive oxygen species, stabilization of mitochondrial membrane potential, and increased antioxidant (superoxide dismutase and catalase) activities. Furthermore, BFO increased the expression of Nrf2, HO-1, and Bcl-2 and decreased the expression of Bax. In conclusion, these findings suggest that BFO protects NRK-52E cells against HG-induced damage by inhibiting apoptosis and oxidative stress through the Nrf2/HO-1 signaling pathway.

## Introduction

Diabetic nephropathy (DN) is the most common chronic kidney disease and a common cause of end-stage renal disease (Johansen et al., 2021). In the past 20 years, the morbidity and mortality of DN have increased significantly in the global population (Bell et al., 2015; Heerspink et al., 2019). The histological features of DN mainly include mesangial expansion, glomerular basement membrane thickening, and podocyte loss (Lin and Susztak, 2016). Currently, the main treatment for DN is to control blood glucose level and blood pressure to delay the development of the disease; however, the effect is not satisfactory (Hsiao et al., 2019). Furthermore, studies that attempted to clarify specific mechanisms leading to the progression of DN remain inconclusive. Although the specific mechanism of DN has not been identified, hyperglycemia is thought to be a potential trigger of renal tubular cell damage (Wang et al., 2017; Zhang YH. et al., 2020; Das et al., 2020).

The occurrence and development of DN are related to oxidative stress and apoptosis caused by hyperglycemia (Chen et al., 2019; Calle and Hotter, 2020). High glucose (HG) can reduce the ability of the cell’s antioxidant enzyme system, increase cell apoptosis rate, and promote reactive oxygen species (ROS) overproduction in renal tubular epithelial cells (He et al., 2015; Lee et al., 2019), thereby causing cell oxidative stress damage. Therefore, regulation of oxidative stress and cell apoptosis is an important approach to attenuate HG-induced injury in renal cells. Nuclear factor erythroid 2–related factor 2 (Nrf2) is a transcriptional regulator and an important cell defense factor (Qaisiya et al., 2014). The Nrf2 signaling pathway plays a critical role in apoptosis and oxidative stress (Nezu et al., 2017) and is also the main antioxidant signaling pathway (Nezu et al., 2017). Nrf2 is considered to be a potential therapeutic target for DN (Tong et al., 2019). Activated Nrf2 can reduce oxidative stress damage, thereby resisting DN *in vivo* and *in vitro* (Zhang et al., 2014; Shin et al., 2019).

Burdock (*Arctium lappa L.*) is a common herb and health supplement in Asia (Gao Q. et al., 2018). Burdock fructooligosaccharide (BFO), a reserve carbohydrate, is a water-soluble inulin-type fructooligosaccharide extracted from burdock root, which consists of a linear chain of *α*-2,1-linked fructofuranose residues with a single *β*-1,2-linked glucopyranose (Hao et al., 2005; Wang Y. et al., 2019). Studies have shown that BFO has a wide range of pharmacological activities. BFO has antioxidant properties and the ability to scavenge free radicals (Jiang et al., 2019), it can significantly regulate lipid metabolism in diabetic rats (Li et al., 2019) and exert antithrombotic effects via regulating the ERK/NF-κB pathway (Qiu et al., 2020), and it has anti-inflammatory effects *in vivo* and *in vitro* (Zhang et al., 2019; Zhang X. et al., 2020), as well as anti-cancer effects (Xu et al., 2019). BFO can also lower fasting blood glucose (FBG) levels and improve glucose tolerance (Gao Y. et al., 2018; Annunziata et al., 2019; Yuan et al., 2021). However, no research has addressed the role of BFO in ameliorating NRK-52E cell apoptosis and oxidative stress injury induced by HG. Therefore, this study aimed to investigate the effects of BFO on NRK-52E cell injury induced by HG.

## Materials and Methods

### Materials and Reagents

Rat renal tubular epithelial cells (NRK-52E cells) were purchased from the National Laboratory Cell Resource Sharing Platform (Beijing, China). Burdock roots were obtained from Yishunkang (Jiangsu, China). The kits for superoxide dismutase (SOD) and catalase (CAT) were acquired from Jiancheng Bioengineering Institute (Nanjing, Jiangsu, China). BCA protein assay kit, ROS assay kit and mitochondrial membrane potential detection kit were acquired from Beyotime Institute of Biotechnology (Haimen, Jiangsu, China). Antibodies specific for Nrf2, HO-1, Bcl-2, Bax, and *β*-actin were obtained from ABclonal (Wuhan, Hubei, China).

### Burdock Fructooligosaccharide Preparation and Fractionation

BFO was isolated and fractionated following our previously reported method (Hao et al., 2005; Yuan et al., 2021). Briefly, burdock roots were submerged in hot water and 95% ethanol for alcohol precipitation. The precipitate was dissolved in distilled water and deproteinized using the Sevag method (Sevag, 1938). The aqueous phase was collected and decolorized using D101 macroporous resin (Solarbio, Beijing, China), followed by loading onto a DEAE-cellulose-52 chromatographic column (Solarbio). The collected fractions were filtered using a 0.22-μm filter membrane, inserted in a 1 kDa regenerated cellulose dialysis bag (Solarbio), and dialyzed at 4°C for 72 h. Then, BFO was further purified by gel filtration chromatography on a Sephadex G75 column (Solarbio) and eluted with distilled water at a flow rate of 0.5 ml/min. The homogeneous fractions from the eluted single peak were gathered, concentrated, and lyophilized to powder (BFO). The homogeneity and molecular weight of BFO were determined by high performance gel permeation chromatography (HPGPC) on a Shimadzu Lc-l0Avp instrument (Shimadzu, Japan) equipped with an Ultrahydrogel™ liner column. Elution was monitored using a Shimadzu RID-10A refractive index detector. A series of standard dextran solutions was run under the same conditions and a standard curve linear over a wide range (1–10 kDa) was obtained by correlation analysis between the dextran standard molar mass and retention time (Liu et al., 2014). The total sugar content of BFO was measured as D-fructose equivalents using the phenol-sulfuric acid method (Dubois et al., 1956). The presence of starch-type polysaccharides was detected using the triiodination reaction (Liu et al., 2018). The Bradford method was adapted to determine the total protein content using bovine serum albumin (BSA) as the standard (Bradford, 1976).

### Cell Culture and Drug Dissolution

NRK-52E cells were maintained in Dulbecco’s modified Eagle’s medium (DMEM) (Gibco, CA, United States) supplemented with 10% fetal bovine serum and 1% penicillin/streptomycin in a humidified atmosphere of 5% CO_2_ at 37°C. The cells were digested and passaged every 1–2 days and seeded into 6- or 96-well plates for experiments. BFO was dissolved in phosphate-buffered saline to prepare the stock solution.

### Cell Viability Assay

NRK-52E cells were cultured in normal glucose (NG, 5.5 mM glucose), HG (30 mM glucose), and HG + different BFO concentrations (62.5, 125, and 250 μg/ml) for 48 h using 96-well plates. Then, CCK-8 kit reagent was added to wells and then the plates were incubated at 37°C for 1–2 h. A microplate reader (Biotek, Winooski, VT, United States) was used to measure the absorbance at 450 nm.

### Cell Apoptosis

NRK-52E cells were subjected to the various culture conditions described above. Thereafter, 5 µL of propidium iodide and 10 µL of Annexin V-fluorescein isothiocyanate (FITC) were added to each sample for 15 min at room temperature (RT) in the dark, followed by the addition of 500 µL binding buffer and filtration through a 300 µm mesh cell filter. Finally, flow cytometry was performed immediately.

### Detection of Intracellular Reactive Oxygen Species Levels

NRK-52E cells were cultured with different substances, as described previously. Samples were then incubated with DCFH-DA for 30 min at 37°C. The percentage of fluorescently positive cells was measured by flow cytometry at 488 nm excitation wavelength and 525 nm emission wavelength.

### Measurement of Mitochondrial Membrane Potential

NRK-52E cells were cultured with different substances, as described previously. Thereafter, JC-1 dyeing working solution was added to the samples, mixed well, and incubated at 37°C for 20 min. At the end of the treatment, JC-1 fluorescence was measured by flow cytometry at 490 nm excitation wavelength and 530 nm emission wavelength.

### Antioxidant System Assay

NRK-52E cells were exposed to different substances, as described previously. The levels of SOD and CAT were determined using relevant detection kits according to the manufacturer’s instructions. At the end of the reaction, a microplate reader was used to measure the absorbance of the samples.

### Western Blotting

NRK-52E cells were incubated with different substances, as described previously. The protein concentration was determined using a BCA protein assay kit, and the denatured protein was separated on SDS-PAGE gel. Protein was transferred to a nitrocellulose membrane under constant current (300 mA) conditions, and then the membrane was blocked with 5% skim milk at RT for 2 h. The membranes were incubated with Nrf2 (1:2000), HO-1 (1:500), Bax (1:1000), and Bcl-2 (1:1000) antibodies at 4°C overnight. Next, the membranes were washed with TBST (3 × 10 min) and then incubated with the secondary antibodies (1:10,000) for 1 h. ECL detection reagent and a chemiluminescence imaging system were used to examine the membranes. The results were analyzed using the ImageJ software.

### RNA Extraction and Quantitative Real-Time PCR

Total RNA was isolated from NRK-52E cells using TRIzol reagent (Beyotime), and cDNA was synthesized from total RNA using Prime Script RT kit (Thermo Fisher Scientific, Waltham, MA, United States) according to the manufacturer’s instructions. GAPDH was used as an internal standard. The primer pairs used for real-time PCR are shown in Table 1. The cycle threshold (Ct) value was determined, and the level of the housekeeping gene GAPDH was used for normalization. The relative mRNA level of each target gene was calculated with the 2^−△△^Ct method.

**TABLE 1:** Primers used for quantitative real-time PCR.

Gene	Forward (5'-3')	Reverse (5'-3')
Nrf2	AAT​TGC​CAC​CGC​CAG​GAC​T	TCA​AAC​ACT​TCT​CGA​CTT​ACC​CC
HO-1	CAG​CAT​GTC​CCA​GGA​TTT​GTC	CCT​GAC​CCT​TCT​GAA​AGT​TCC​TC
Bax	ATG​GGC​TGG​ACA​CTG​GAC​TT	TTC​CAG​ATG​GTG​AGT​GAG​GCA
Bcl-2	TTG​TGG​CCT​TCT​TTG​AGT​TCG	GCA​TCC​CAG​CCT​CCG​TTA​T
GAPDH	CTG​GAG​AAA​CCT​GCC​AAG​TAT​G	GGT​GGA​AGA​ATG​GGA​GTT​GCT

### Statistical Analysis

Statistical analysis was conducted with SPSS 22.0. Data are shown as the mean ± standard deviation. Differences among groups were compared by one-way analysis of variance, followed by Dunnett’s post hoc test. *p* < 0.05 was considered statistically significant.

## Results

### Isolation and Purification of Burdock Fructooligosaccharide

**FIGURE 1:**
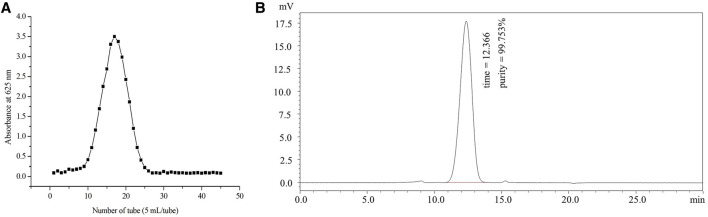
Preparation and fractionation of BFO. **(A)** Elution curve of gel filtration chromatography on a Sephadex G75 column. **(B)** Molecular weight and homogeneity of BFO determined by HPGPC.

The elution curve of gel filtration chromatography on a Sephadex G75 column presented BFO as a single component (Figure 1A). The total sugar content of BFO was determined to be 99.7%. After concentration and lyophilization, BFO presented as a white powder and tested negative for the triiodide reaction, indicating the absence of starch-type polysaccharides. The Bradford test result was negative with no absorption at 280 or 260 nm, suggesting the absence of proteins and nucleic acids in BFO. The homogeneity and molecular weight of BFO were determined by high-performance gel permeation chromatography (HPGPC). The retention time and purity of BFO were 12.366 min and 99.753%, respectively. BFO presented a single and symmetrically sharp peak (Figure 1B), indicating a homogenous fraction with a molecular weight of 3,194 Da.

### Burdock Fructooligosaccharide Increased the Cell Viability of NRK-52E Cells Under High Glucose Condition

**FIGURE 2:**
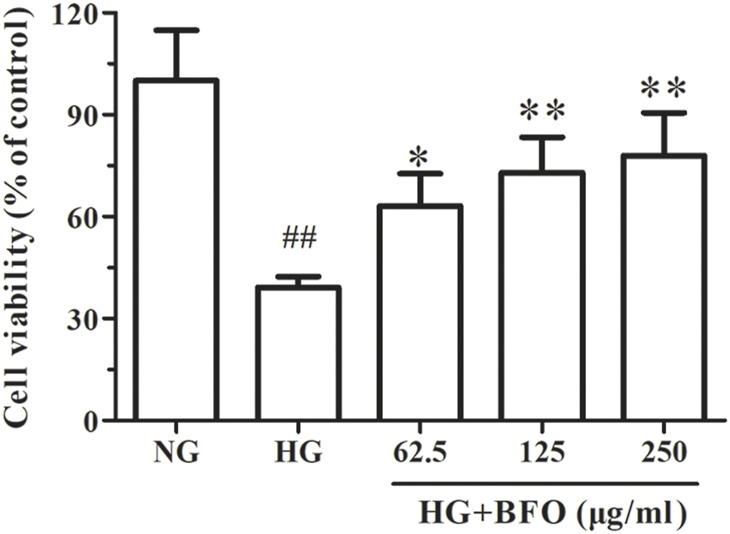
Effect of BFO on the viability of NRK-52E cells. The cell viability was determined by a Cell Counting Kit-8 (CCK-8) test of NRK-52E cells cultured with high glucose (HG) and different concentrations of BFO. All data are expressed as the mean ± SD. ^##^ *p* < 0.01 vs. normal glucose (NG), ^*^ *p* < 0.05 vs. HG, ^**^ *p* < 0.01 vs. HG.

The CCK-8 assay was conducted to investigate the effect of BFO on the viability of NRK-52E cells under HG condition. The cell viability of the HG group was significantly reduced compared to that of the NG group. Conversely, when NRK-52E cells were incubated with HG + different BFO concentrations (62.5, 125, and 250 μg/ml), the cell viability increased (63.16, 72.97, and 77.98% of the control value, respectively) (Figure 2).

### Burdock Fructooligosaccharide Reduced the Apoptosis Rate of NRK-52E Cells Under High Glucose Condition

**FIGURE 3:**
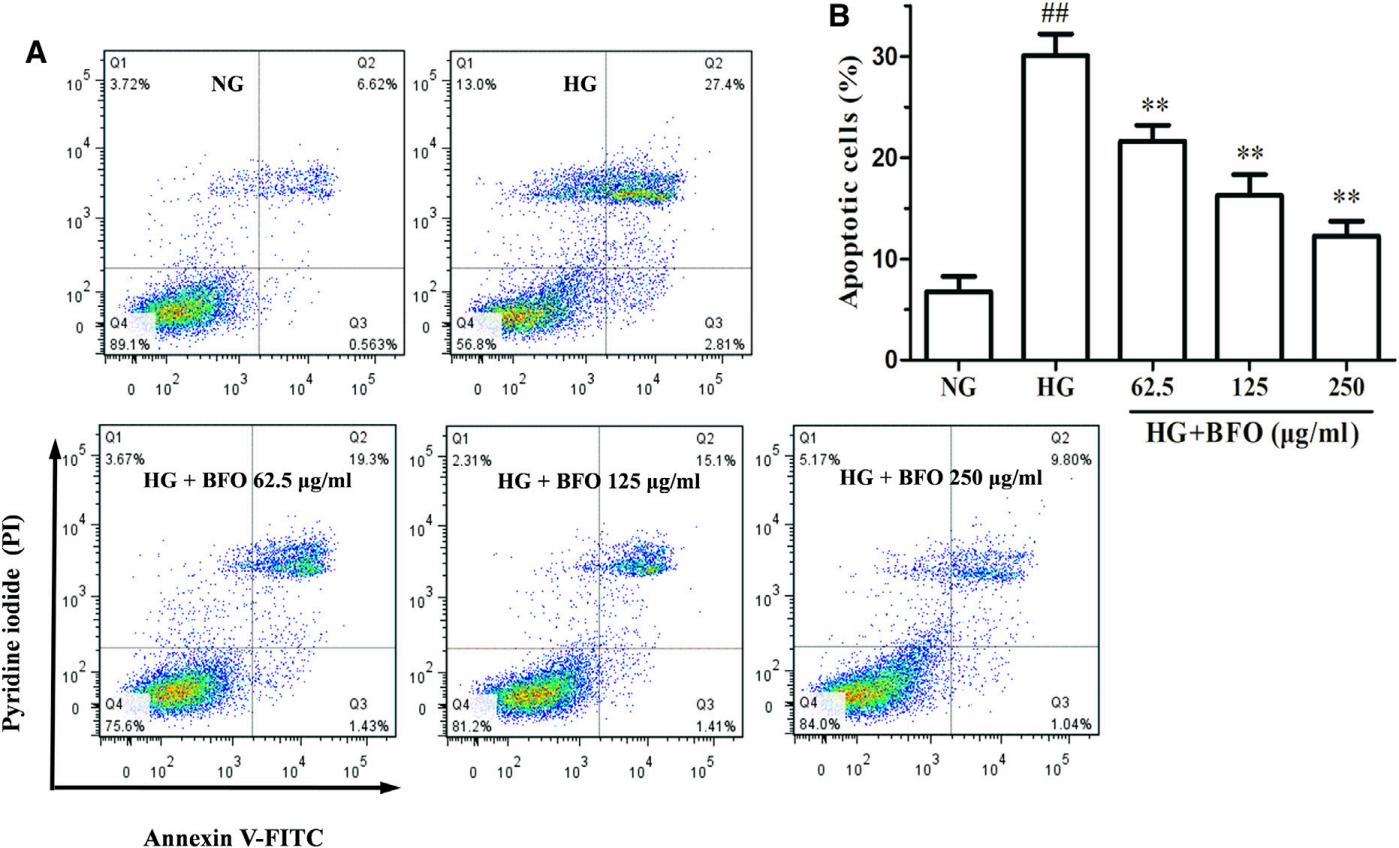
Effect of BFO on apoptosis in NRK-52E cells. Apoptosis of NRK-52E cells cultured with high glucose (HG) and different concentrations of BFO was measured by Annexin V-FITC/PI staining. **(A)** Representative images of apoptotic NRK-52E cells determined *via* flow cytometry. **(B)** Quantification of apoptotic NRK-52E cells. All data are expressed as the mean ± SD. ^##^ *p* < 0.01 vs. normal glucose (NG), ^**^ *p* < 0.01 vs. HG.

To study the effect of BFO on the apoptosis rate of NRK-52E cells under HG condition, the cell apoptosis rate was measured by flow cytometry. The apoptosis rate of the cells cultured with NG, HG, and HG + different BFO concentrations (62.5, 125, and 250 μg/ml) were 7.183 ± 1.230; 30.210 ± 1.741; and 20.719 ± 1.280, 16.510 ± 1.697, and 10.84 ± 0.172%, respectively. The apoptosis rate of the HG group was significantly higher than that of the NG group. Conversely, compare with HG, the intervention with HG + different BFO concentrations significantly reduced the cell apoptosis rate (Figure 3).

### Burdock Fructooligosaccharide Reduced the ROS Level of NRK-52E Cells Under High Glucose Condition

**FIGURE 4:**
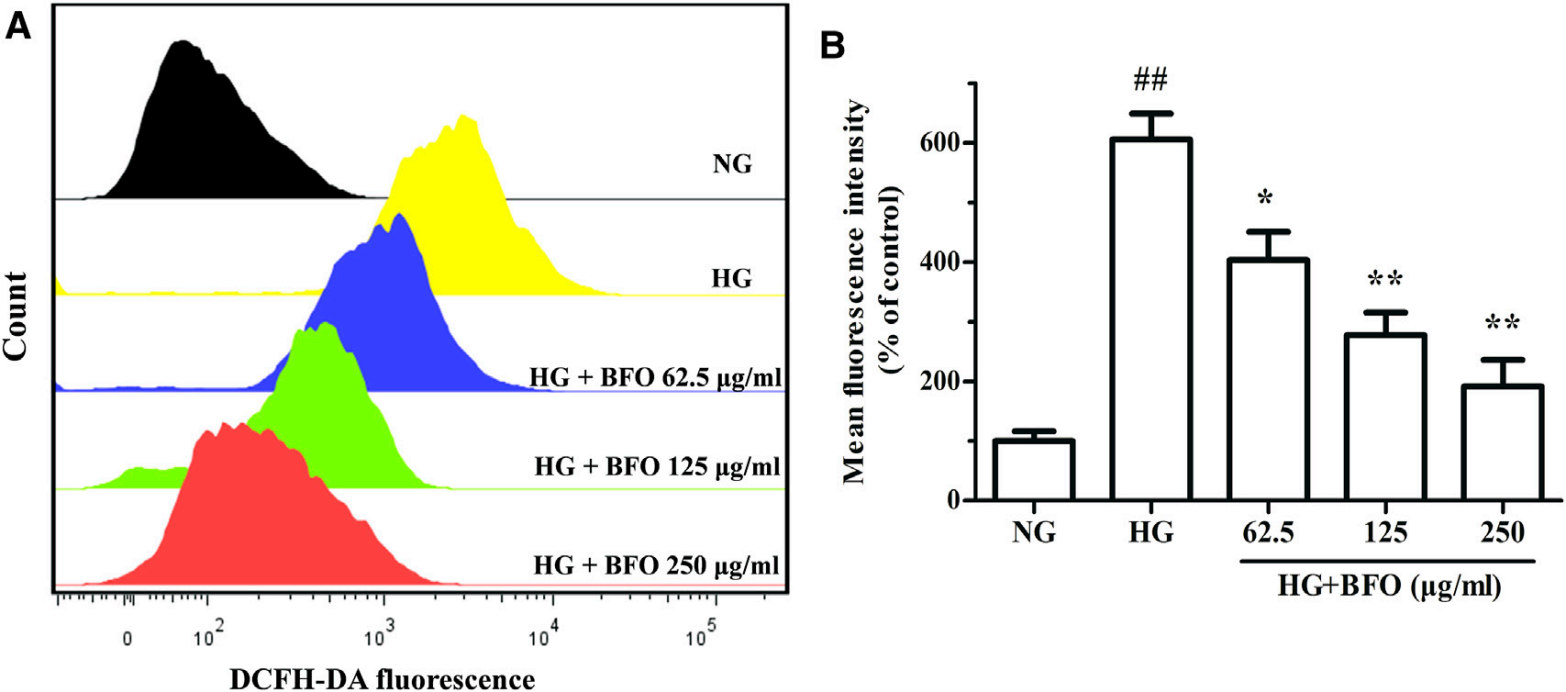
Effect of BFO on the level of reactive oxygen species (ROS) in NRK-52E cells. **(A)** Representative images of ROS generation measured by flow cytometry using the DCFH-DA probe. **(B)** Histogram representing the quantitative analysis of ROS accumulation in NRK-52E cells. All data are expressed as the mean ± SD. ^##^ *p* < 0.01 vs. normal glucose (NG), ^*^ *p* < 0.05 vs. HG, ^**^ *p* < 0.01 vs. HG.

The DCFH-DA method was used to measure ROS level in NRK-52E cells exposed to HG and HG + different BFO concentrations. The results indicated that the ROS level of the HG group was higher than that of the NG group. Compare with HG, the intervention with HG + different BFO concentrations significantly reduced the level of ROS in a dose-dependent manner (Figure 4).

### Burdock Fructooligosaccharide Decreased the Mitochondrial Membrane Potential of NRK-52E Cells Under High Glucose Condition

**FIGURE 5:**
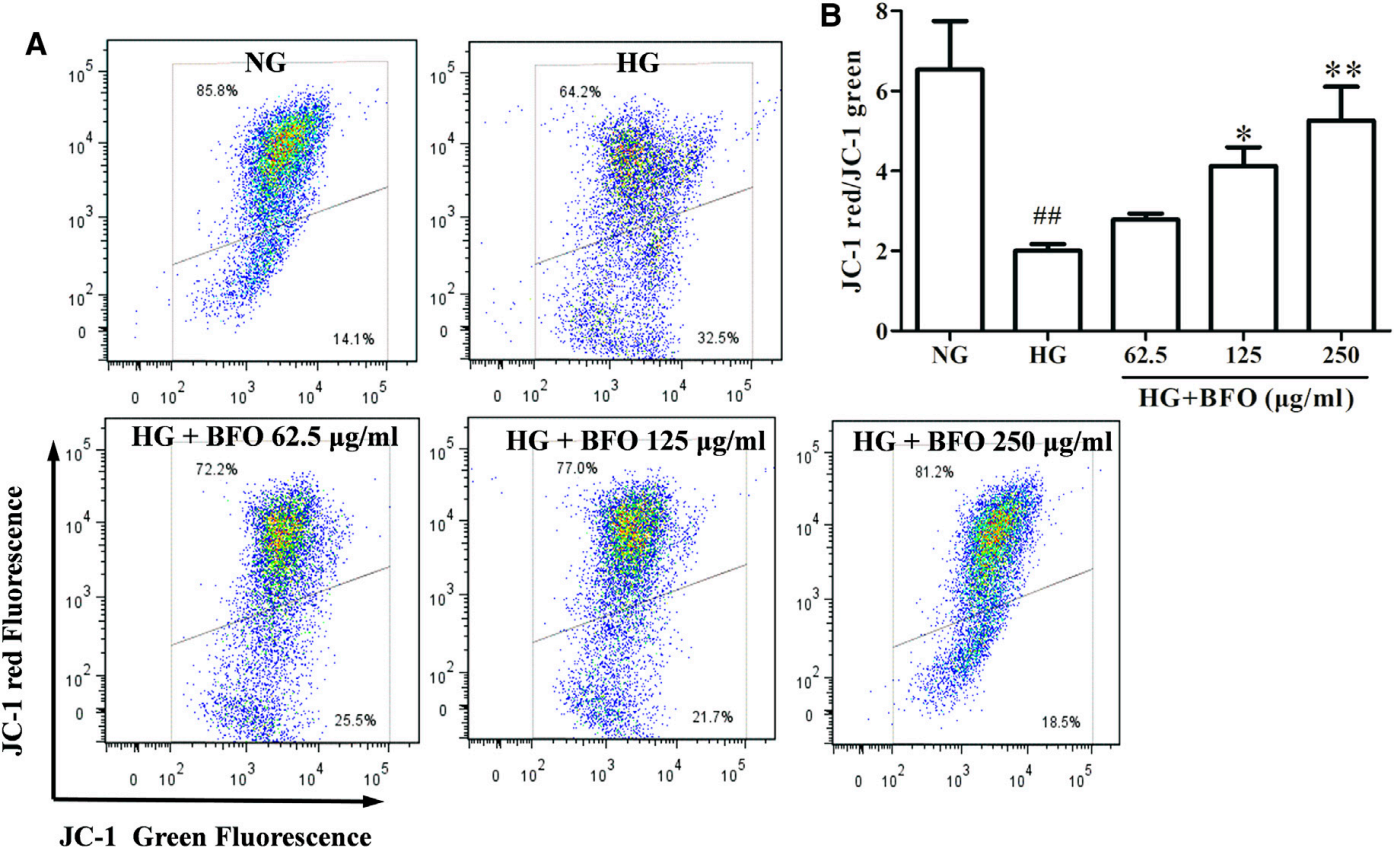
Effect of BFO on mitochondrial membrane potential in NRK-52E cells. **(A)** Representative images of mitochondrial membrane potential determined by flow cytometry using the JC-1 probe. **(B)** Histogram representing the quantitative analysis of mitochondrial membrane potential in NRK-52E cells. All data are expressed as the mean ± SD. ^##^ *p* < 0.01 vs. normal glucose (NG), ^*^ *p* < 0.05 vs. HG, ^**^ *p* < 0.01 vs. HG.

To elucidate the effect of BFO on the mitochondrial membrane potential in NRK-52E cells under HG condition, the mitochondrial membrane potential was determined by flow cytometry. The results demonstrated that in the HG group, the mitochondrial membrane potential was significantly decreased (Figure 5). The mitochondrial membrane potential of cells treated with HG + 62.5 μg/ml BFO did not increase significantly, and BFO at 125 and 250 μg/ml effectively inhibited the decrease in cell mitochondrial membrane potential induced by HG.

### Burdock Fructooligosaccharide Increased the Levels of Superoxide Dismutase and Catalase in NRK-52E Cells Under High Glucose Condition

**FIGURE 6:**
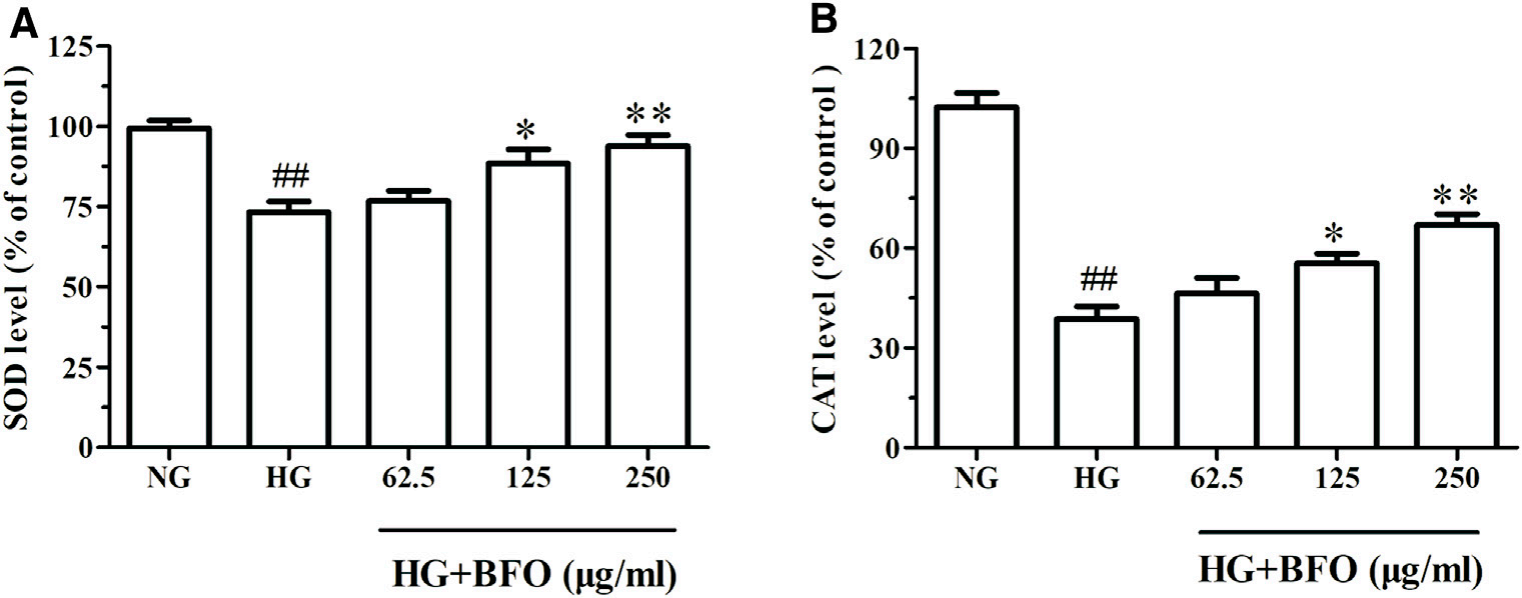
Effect of BFO on SOD **(A)** and CAT **(B)** levels in NRK-52E cells. All data are expressed as the mean ± SD. ^##^ *p* < 0.01 vs. normal glucose (NG), ^*^ *p* < 0.05 vs. HG, ^**^ *p* < 0.01 vs. HG.

To elucidate whether BFO could protect NRK-52E cells against HG-induced oxidative stress damage, the activities of SOD and CAT were detected. The activities of SOD and CAT in the HG group were significantly lower than those in the NG group; both 125 μg/ml and 250 μg/ml BFO effectively inhibited the HG-induced reduction in SOD and CAT levels (Figures 6A,B).

### Burdock Fructooligosaccharide Affected Nrf2, HO-1, Bax, and Bcl-2 Protein and mRNA Expression in NRK-52E Cells Under High Glucose Condition

**FIGURE 7:**
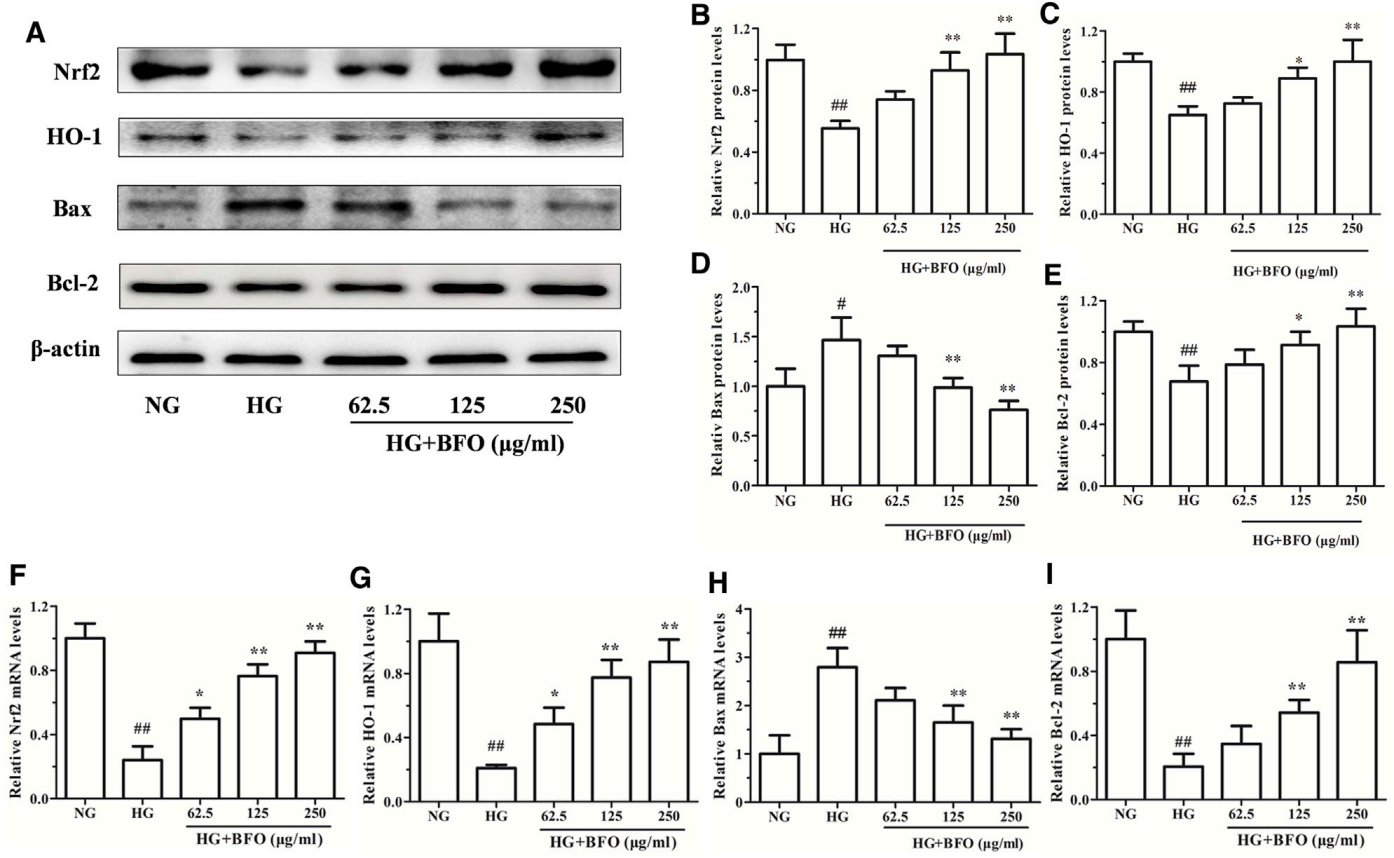
Effect of BFO on the protein and mRNA expression in NRK-52E cells. **(A)** Representative bands of Nrf2, HO-1, Bax, and Bcl-2 obtained by western blotting. Relative protein levels (band density) of Nrf2 **(B)**, HO-1 **(C)**, Bax **(D)**, and Bcl-2 **(E)** were calculated by Image J. Relative mRNA levels of Nrf2 **(F)**, HO-1 **(G)**, Bax **(H)**, and Bcl-2 **(I)** in NRK-52E cells were determined by real-time PCR. All data are expressed as the mean ± SD. ^#^ *p* < 0.05 vs. normal glucose (NG), ^##^ *p* < 0.01 vs. NG, ^*^ *p* < 0.05 vs. HG, ^**^ *p* < 0.01 vs. HG.

To observe the molecular mechanisms underlying the protective effects of BFO on NRK-52E cells, the protein and mRNA expression of Nrf2, HO-1, Bax, and Bcl-2 in NRK-52E cells induced by HG was determined by western blotting and real-time PCR, respectively. The protein levels of Bax in the HG group were significantly increased compared to those in the NG group, and the protein levels of Nrf2, HO-1, and Bcl-2 in the HG group were significantly decreased. In addition, the protein levels of Bax in the HG + BFO group were significantly decreased in a dose-dependent manner, and the protein levels of Nrf2, HO-1, and Bcl-2 in the HG + BFO group were significantly increased (Figures 7A–E). Furthermore, the mRNA expression of Nrf2, HO-1, Bax, and Bcl-2 in these groups was in accordance with the protein expression (Figures 7F–I).

## Discussion

In this study, we aimed to study the protective effect of BFO on NRK-52E cell apoptosis and oxidative stress induced by HG. Although the exact mechanism of HG-induced renal tubular epithelial cell injury has not yet been fully clarified, researches have shown that oxidative stress and apoptosis play important roles in the development and pathogenesis of DN (Tiong et al., 2019; Lee et al., 2020). NRK-52E cells cultured with HG are widely used as a DN model (Hou et al., 2014; Slyne et al., 2015). In addition, renal tubular epithelial cell injury is the main factor leading to DN. Some studies have shown that some plant polysaccharides can improve the oxidative stress damage caused by diabetes and its complications (Liao et al., 2019; Yang et al., 2020). BFO is a plant polysaccharide extracted from burdock that has been reported to exert antidiabetic effects (Yuan et al., 2021). Thus, we examined the effect of BFO on NRK-52E cell injury induced by HG and demonstrated that BFO has a protective effect on oxidative stress damage and apoptosis induced by HG *in vitro*. The protective effects of BFO were related to the inhibition of ROS, increase in mitochondrial membrane potential as well as CAT and SOD levels**,** and regulation of Bcl-2 and Bax protein expression. Furthermore, we also found that these protective effects depended on the regulation of the Nrf2/HO-1 signaling pathway.

Renal tubular epithelial cells undergo oxidative stress and apoptosis under HG, hypoxia, and other environments, leading to tubular interstitial fibrosis and even kidney failure in severe cases (Yao et al., 2017). Studies have found that under HG conditions, renal tubular epithelial cell viability is reduced, and apoptosis rate is increased (Lv et al., 2019). The DN model was constructed by culturing NRK-52E cells in DMEM medium containing 30 mM glucose. CCK-8 and Annexin V-FITC/PI double staining assays were used to measure the cell viability and cell apoptosis rate. Our results provided evidence that BFO significantly increased cell viability and decreased the apoptosis rate at 62.5–250 µg/ml concentrations at 48 h in an HG environment.

It has been reported that HG-induced oxidative stress in renal tubular epithelial cells plays a critical role in the pathogenesis of DN (Xie et al., 2019). Mitochondria are the main source of oxygen and ROS (Sinha et al., 2013). When mitochondria are challenged by hyperglycemia, the mitochondrial membrane potential is affected, resulting in decreased mitochondrial membrane potential and increased ROS levels (Chen et al., 2018). Overproduction of ROS is known to be a major cause of oxidative stress and cell apoptosis (Volpe et al., 2018). Furthermore, ROS have been reported to play a key role in NRK-52E cell oxidative damage induced by HG (Tsikas, 2017). SOD and CAT are essential antioxidant enzymes in humans and animals that protect against oxidative stress (Altintas et al., 2010; Kehrer and Klotz, 2015). When the antioxidant defense system is destroyed, ROS production increases, thereby leading to ROS accumulation (Jha et al., 2016). Additionally, HG could result in oxidative injury, which eventually leads to the overproduction of ROS impairing cellular antioxidant systems (CAT and SOD) (Tong et al., 2018). Our current findings demonstrated that HG significantly decreased the mitochondrial membrane potential and resulted in ROS overproduction. In addition, the levels of SOD and CAT were decreased under HG conditions in NRK-52E cells. However, this change was partially reversed by BFO treatment in NRK-52E cells. These results proved that BFO might play a role in NRK-52E cell oxidative stress damage induced by HG.

Apoptosis is a type of programmed cell death that mainly occurs through the death receptor pathway and mitochondrial pathway (Xie et al., 2020). The Bax/Bcl-2 gene plays a key role in the process of apoptosis, and the pathway associated with it is the key pathway of cell apoptosis (Peña-Blanco and García-Sáez, 2018). Hyperglycemia can activate the apoptotic pathway and regulate the expression of apoptotic proteins, which play an important role in HG-induced renal tubular epithelial apoptosis (Wang Z. et al., 2019). Nrf2 is a transcriptional regulator, and under normal conditions, it binds to Keap1 in the cytoplasm. After external stimulation, Nrf2 dissociates from Keap1 and enters an activated state. Nrf2 enters the nucleus from the cytoplasm, regulates its downstream-related factors, and exerts an antioxidant effect (Eve et al., 2016; Figueroa and Wright, 2016). HO-1 is a downstream protein of Nrf2, which promotes the degradation of hemoglobin, effectively reducing inflammation and oxidative stress damage, thereby protecting cells (Humphries et al., 2016; Feng et al., 2019). Several researches have confirmed that the expression of Nrf2 and HO-1 proteins in renal tubular epithelial cells is downregulated by HG and that protein expression increases after administration of BFO (Zhou et al., 2019; Lu et al., 2020). To investigate the molecular mechanism underlying the effect of BFO in ameliorating NRK-52E cell oxidative stress and apoptosis *in vitro*, the protein expression of Bax, Bcl-2, Nrf2, and HO-1 in NRK-52E cells was detected. Bax expression increased after HG stimulation, and Bcl-2, Nrf2, and HO-1 expression decreased. BFO treatment reversed the effect of HG on the expression of Bax, Bcl-2, Nrf2, and HO-1, and prevented HG-induced oxidative stress and apoptosis in NRK-52E cells. Therefore, we speculate that BFO can prevent oxidative stress and apoptosis in renal tubular epithelial cells under HG conditions and that Nrf2-HO-1 plays an important role in NRK-52E cell damage induced by HG.

In conclusion, our experiments demonstrated that BFO increased cell viability and attenuated cell apoptosis and oxidative damage induced by HG in NRK-52E cells, and that the effect might be mediated through the Nrf2/HO-1 signaling pathway. Taken together, our results indicate that BFO may be an effective treatment for DN in clinical practice. However, the results of *in vitro* studies do not guarantee that BFO plays a similar role *in vivo*. In addition, the HG-induced diabetic nephropathy model is considerably different from naturally occurring diabetic nephropathy. Before clinical application, further in-depth research is needed to evaluate the anti-diabetic nephropathy effects of BFO.

## Data Availability

The raw data supporting the conclusion of this article will be made available by the authors, without undue reservation.
